# Facile ultrasonic preparation of a polypyrrole membrane as an absorbent for efficient oil-water separation and as an antimicrobial agent

**DOI:** 10.1016/j.ultsonch.2021.105746

**Published:** 2021-09-04

**Authors:** Moorthy Maruthapandi, Arumugam Saravanan, Shanmugasundaram Manoj, John H.T. Luong, Aharon Gedanken

**Affiliations:** a Department of Chemistry, Bar-Ilan Institute for Nanotechnology and Advanced Materials, Bar-Ilan University, Ramat-Gan 52900, Israel; b School of Chemistry, University College Cork, Cork T12 YN60, Ireland

**Keywords:** Polypyrrole, Ultra-sonication coating, Oil absorption, Antibacterial activity

## Abstract

Polypyrrole (PPY) spherical particles synthesized using carbon dots as an efficient catalyst were strongly embedded on fluorinated nonwoven fabric by ultrasonication to form a membrane with high hydrophilicity. An optimal amount of PPY adhered to the membrane after 30 min of sonication enhanced the overall membrane area with high hydrophilicity. Oil with high hydrophobicity was repelled by the resulting membrane, whereas water was freely penetrated and diffused from the membrane. The membrane exhibited good reusability and efficiency for the recovery of oil from a cooking oil–water mixture within 30 s. The incorporation of PPY in the fluorinated fabric imparts significant antibacterial properties against two common pathogens, *Escherichia coli* (Gram-negative) and *Staphylococcus aureus* (Gram-positive). The anti-biofouling membrane could pave the way for its potential application to separate spilled oil from contaminated waters, comprising different microorganisms and living species. The novelty of this manuscript is described in a new system, the fabrication of PPY membranes with two important properties: biocidal and oil/water separation.

## Highlights

• Synthesis of polypyrrole from pyrrole involves only carbon dots as the catalyst.
• PPY particles are optimally deposited on fluorinated nonwoven fabric by ultrasonication after 30 min.
• With enhanced hydrophilicity, the resulting membrane can separate oil from a mixture of oil–water within 30 s with good reusability and efficiency.
• The incorporation of PPY in the fluorinated nonwoven membrane also imparts superior antibacterial properties against *E. coli* (Gram-negative) and *S. aureus* (Gram-positive).

## 1 Introduction

Oil spill incidents occur during storage, transportation, and extraction, which are considered major environmental issues with serious long-lasting health and ecological effects. Considering the frequent oil spill worldwide, it is of uttermost importance to isolate and separate oily wastewater from the ocean, lakes, and river waters [1], [2], [3]. Various chemical, biological, and mechanical processes have been explored for dealing with oil–water contamination, e.g., filtration [4], degradation [5], skimming [6], in situ burning [7], and separation [8], [9]. A fast and simple step to remove the bulk oil is an important route to avoid further environmental pollution and the spread of contaminated water. Among such methods, the absorption process via polymer materials has been considered applicable due to its straightforward operation, no secondary water contamination, and energy conservation [10], [11], [12], [13], [14]. However, traditional natural and inorganic materials have a rather insufficient recyclability and low absorption capacity. Hydrophobic polymer materials have attracted considerable attention due to their high surface area and low density. Of note for the water–oil separation are cross-linked polymer materials [15], [16], coated polymer membranes [2], [17], chitosan [18], [19], [20], and cellulose materials [21], [22] with good porous and absorption properties. However, the diffusion of highly viscous oil into such polymer materials is sluggish, resulting in inefficient oil removal. Polymer-coated filters provide an inexpensive, environmentally friendly, and effortless way to separate oil from contaminated water without chemical additives. Hydrophilic pores in a polymer-coated membrane are smaller compared to the oil droplet, i.e., water can easily infuse through the membrane. In contrast, oil droplets remain on the membrane due to their larger size and the repulsive capillary force applied via the pores [23], [24], [25], [26]. Polypyrrole (PPY) [2], [27], [28] is a versatile polymer with remarkable optical, electronic, and magnetic properties over semiconductors or metals. Its other distinct features include flexibility, adjustable electrical conductivity, ease of processing, and low toxicity [24], [29], [30], [31].

The paper unravels a facile, eco-friendly, and ultrasonication process for coating PPY particles on fluorinated nonwoven fabric. PPY particles can be easily synthesized by carbon dot-initiated polymerization methods [32], [33], [34], [35]. Polypyrrole/silver nanoparticles (PPy/AgNPs) have been loaded onto spandex fabric for separating the water and oil. However, PPY has not been used to modify fluorinated nonwoven fabric for oil–water separation [36]. The ultrasonication makes sound energy for the activation and deposition of PPY on the membrane. Apart from the sonication amplitude, the only controlled parameter is the sonication time that is optimized to attain the maximum PPY amount coated on the membrane. The PPY-coated membrane is demonstrated as an absorbent to separate oil and other organic chemicals from water. The antibacterial activity of the PPY membrane is also systematically investigated against *E*. *coli* and *S. aureus* toward the development of a new class of membrane with anti-fouling properties.

## 2 Experimental methods

### 2.1 Materials

The fluorinated non-woven fabric material was commercially obtained from FILC d.o.o., Trata 48, Škofia Loka, Slovenia (https://www.filc.si/en/). Cooking oil and vacuum oil were purchased from a local market. Toluene, petroleum ether, chloroform, and dimethyl sulfoxide were supplied by Sigma Aldrich, Israel.

### 2.2 Synthesis of PPY

0.8-g PPY and 3 mL of (carbon dots) CDs were added to a round bottom flask having H_2_SO_4_ and HCl (1 M 1:2 ratio). The reaction mixture was allowed for 3 min of microwave irradiation, resulting in a deep brown solid. The precipitated polymer was filtered, washed 3 times with double distilled water (DDW), and dried under vacuum. [32]

### 2.3 Contact angle measurement

A Rame–Hart goniometer was used to determine the apparent contact angle of the reported membranes (Model 500). A micro-syringe was used to delicately deposit 10-mL drops of bi-distilled water onto the surfaces of membranes, and the contact angle was recorded. The mean apparent contact angle on membrane surfaces was calculated using 6 measurements.

### 2.4 Instruments and characterization

An FEI Magellan 400 L microscope was used to examine the morphology of PPY-coated fabric (FEI, Hillsboro, OR, USA). The XRD pattern was analyzed using an X-ray diffraction technique on a Bruker AXS D8 Advance diffractometer. A Transon 27 spectrometer was used to acquire FTIR spectra (Bruker, Bremen, Germany). A Nexsa X-ray photoelectron spectrometer (XPS) was used for X-ray photoelectron spectroscopy (Thermo Fisher, England). High-resolution scanning electron microscopy (HRSEM) using an FEI Megallon 400 L microscope. For HRSEM, the sample was prepared by placing a small piece of the material on a carbon tape with a copper plate and it was coated with Au to avoid the charging effect.

## 3 Result and discussion

### 3.1 Ultrasonic-assisted coating of PPY on fabric

**Scheme 1:**
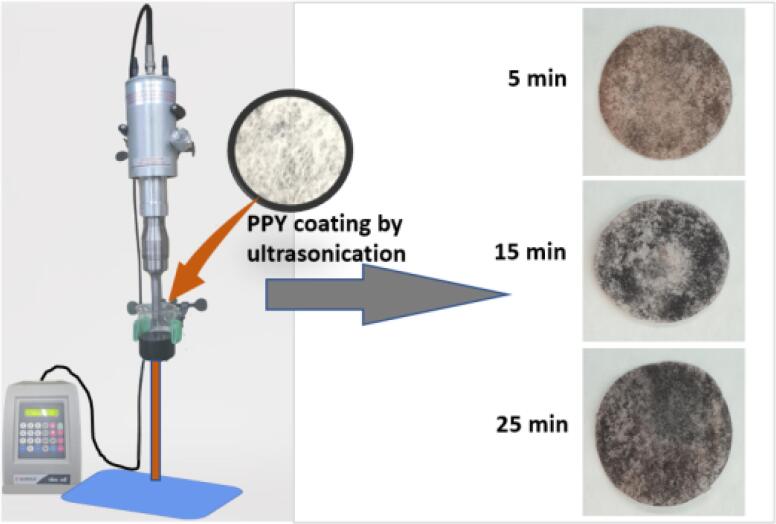
Ultrasonication-assisted coating of PPY particles on fluorinated nonwoven fabric.

The ultra-sonication makes sound energy that activates the PPY to deposit it on the membrane in the solution. Acoustic cavitation is the process that includes the formation, growth, and implosive collapse of the gas bubbles that are formed in the liquid. The bubbles are formed due to the decrease in the intermolecular forces by the presence of invisible particles or gas bubbles. The formed bubble grows by diffusion of solute vapor to the volume of the bubble. The localized hotspots forming extreme conditions that reduce the PPY particle consequently stimulating the small-sized PPY particle to place on the membrane by the sonicating system. Ultrasound cavitation forms a strong interaction between the PPY and membrane. Ultrasound cavitation in liquid–solid systems creates shockwaves and microjets that are directed toward the solid surface and accelerates the PPY particles to higher velocities, which can lead to strong direct deposition on a membrane and forming strong adsorbent material. In brief, 0.3-g PPY was dispersed in 80-mL DDW and sonicated for 1 min for the broad dispersion of PPY particles. The membrane (fluorinated non-woven fabric material) was introduced in the solution and the reaction mixture was kept under sonication for different times (Table 1) with a 35% amplitude. It is worth mentioning that particles were strongly embbeded in the fabric, which could not be removed by simple washing. The sonochemical coating of cotton withstands 65 washing cycles at hospital-washing standards and retains its antibacterial properties after sonication [37]. In this work, the sonochemical method was used to deposit the PPY on the fabric and washed several times with double-distilled water, and dried at 60 °C (Scheme 1). The weight of the membrane was recorded (before and after coating) to estimate the quantity of PPY particles on the membrane (Table 1).

**Table 1:** The quantity of PPY particles on fabric with different sonication times.

Material	Sonication time (min) with 35% of the amplitude	Coated number of PPY particles on fabric (g)	Particle size (nm)
PPYM1	5	0.024	50–500
PPYM2	15	0.062	
PPYM3	30	0.087

### 3.2 Oil/water separation method

All oil–water experiments were conducted by 10 mL of various oil/organic solvents including (corn oil, vacuum oil, toluene, petroleum ether, chloroform, and dimethyl sulfoxide). Oil in water with a volumetric ratio (2:1) in a 50-mL flask and the PPY membrane were positioned under the flask to filter oil from the water. The weight variation was observed (before and after) oil absorption to calculate the absorption capacity. The absorption capacity was conducted by three wt.% PPY-coated membranes. The oil absorption experiment was also conducted in the seawater. The absorption capacity (*AC*) is analyzed as (*W*_f_-*W*_i_)/*W*_i_ where *W*_i_ and *W_f_* are the weight of the PPY membrane before and after oil absorption. At equilibrium, the collection capacity (*CC*) is calculated as (*W*_f_-*W*_i_)/*W*_t_ with *W*_t_ as the total amount of oil in the oil–water mixture. The volumetric flux (*F*_o_, Lm^-2^h^-1^atm^−1^) of oil is then calculated as *V*_o_/*A*_)_ *pt*_s_, where *A* is the active area, *t*_s_ = separation time (s), and *p* = atmospheric pressure (atm). The separation efficiency (η) is defined as (*V*_i_/*V*_f_) *100, where *V*_i_ is the initial oil volume and *V*_f_ is the final oil volume after separation.

**Fig. 1:**
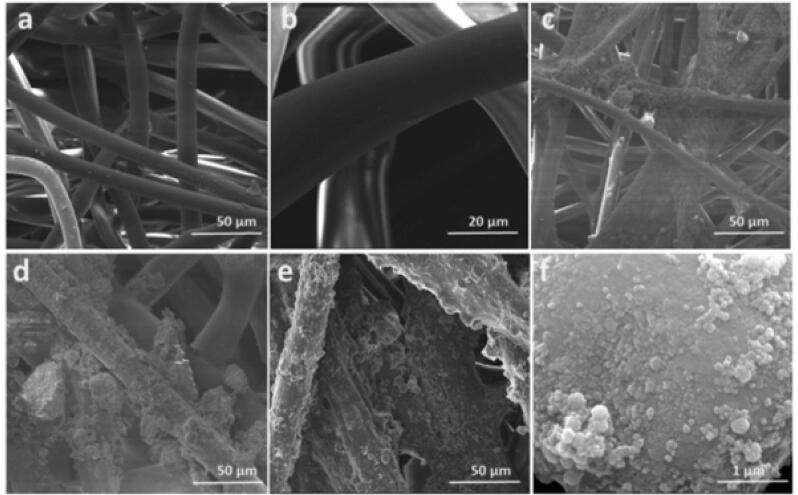
Microscopic morphological images of fibers. Low and high magnification SEM images of (a and b) Membrane (c-f) PPY on fabric.

SEM morphological studies confirmed the different wt% of PPY particles on the membrane as a function of the sonication time. The micro tube-sized membrane without coating with different magnifications of the SEM image is shown in Fig. 1a, b. The deposition of PPY particles on the membrane wall by ultrasonication with various sonication times is illustrated in Fig. 1 c-f. The amount of particle deposition increased with increasing sonication time with a sizable number of PPY particles ranging from 50 to 1000 nm after 30 min of sonication (Fig. 1e). Of note was the use of an amplitude of 35% of the probe, which was needed to reduce the PPY particle sizes during sonication.

**Fig. 2:**
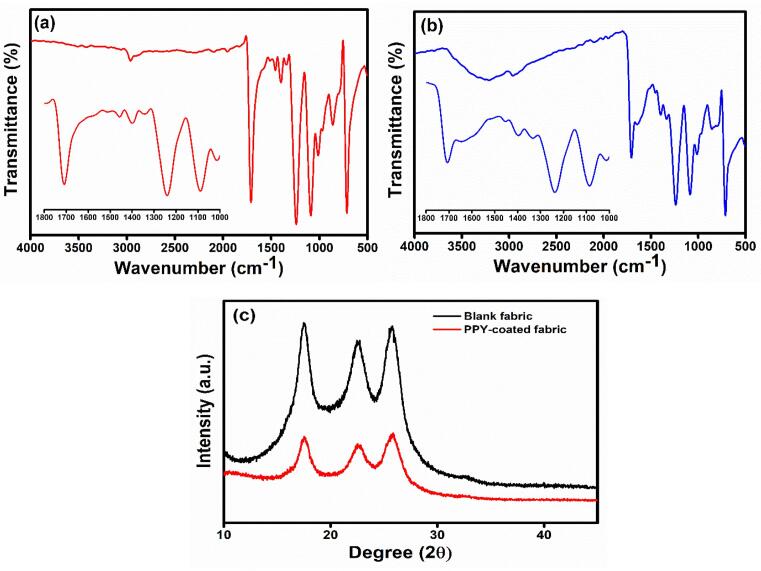
FTIR spectra of blank fabric (a), PPY-coated fabric (b), and XRD pattern of blank fabric and PPY-coated fabric (c).

The functional groups of blank fabric and PPY-coated fabric were characterized with FTIR (Fig. 2a and b). The blank fabric shows three major sharp peaks at 1710 cm^−1^ , which were assigned to C = O stretching (non-conjugated carbonyl) [38]. The peaks at 1240 and 1088 cm^−1^ were accredited to C-O stretching. The lower peaks located at 1398 and 1456 cm^−1^ were assigned to C–H deformation. The peak at 2960 cm^−1^ was allotted to the aliphatic C–H of fabric. While in the case of PPY coated fabric, the peaks at 3213 and 2960 cm^−1^ were assigned to N–H stretching of PPY and aliphatic C–H of the blank fabric. The other peaks at 1706 and 1645 cm^−1^ were attributed C = O for non-conjugated carbonyl and conjugated carbonyl stretching vibration.[38]. Fig. 2c shows X-ray diffraction (XRD) of blank fabric and PPY coated fabric. The diffraction peaks at 17°, 22.5° and 25.7° were related to the fabric surface. The concentration of PPY on the surface of fabric is very less (24 mg), compared to pristine PPY. There were no obvious changes and crystallinity in the XRD pattern of blank and PPY-coated sample.

**Fig. 3:**
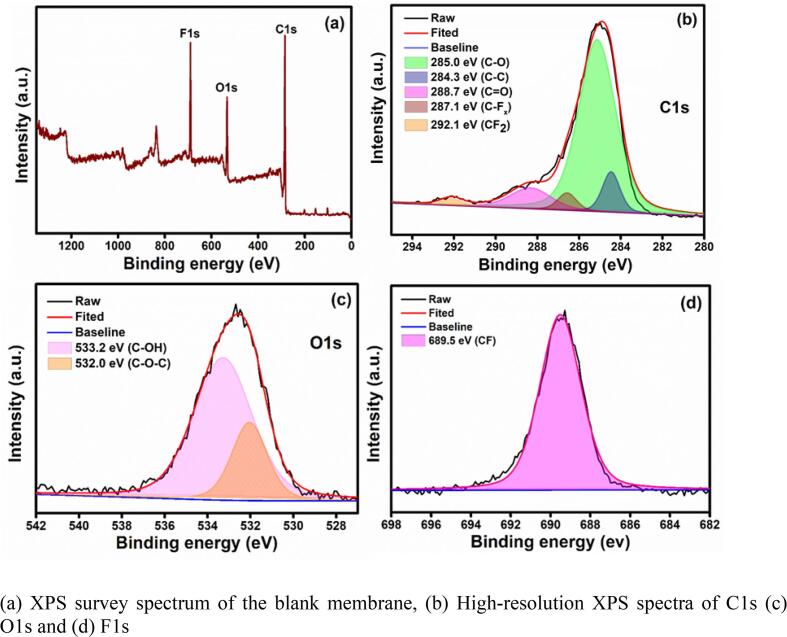
(a) XPS survey spectrum of the blank membrane, (b) High-resolution XPS spectra of C1s (c) O1s and (d) F1s.

**Fig. 4:**
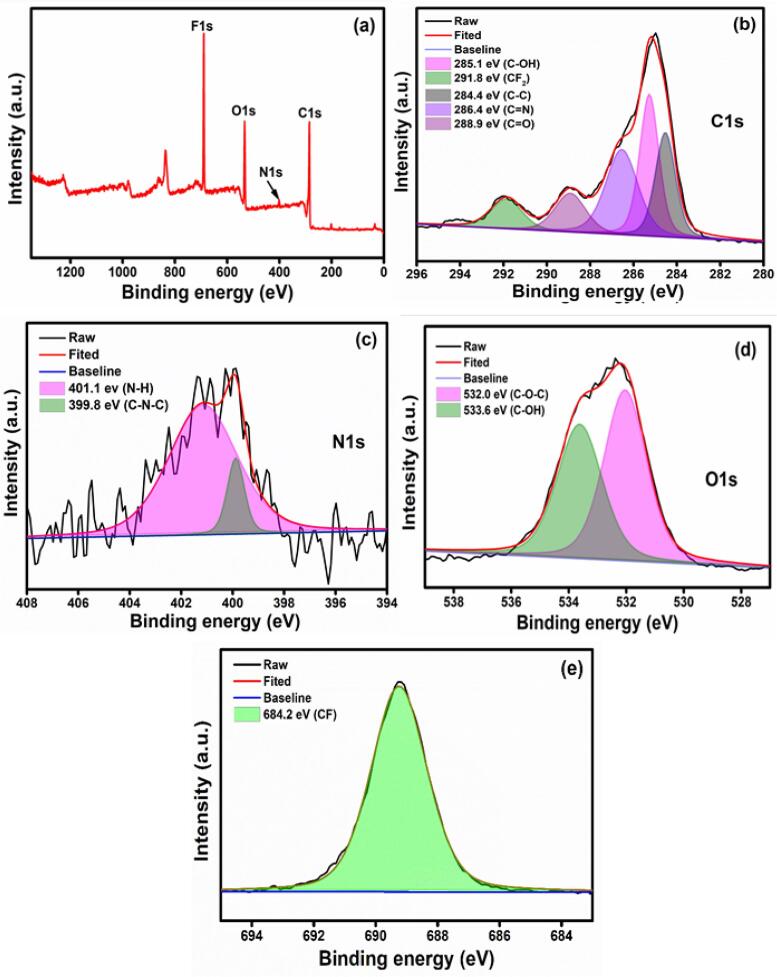
(a) XPS survey spectrum of the PPY coated membrane, (b) High-resolution XPS spectra of C1s (c) N1s, (d) O1s, and (e) F1s.

XPS analysis confirmed the presence of key chemical compositions in the prepared samples. The full XPS survey spectrum of the blank membrane in Fig. 3a shows the presence of C1s, O1s, and F1s in the pristine membrane. The nitrogen element was observed in the PPY-coated membrane reveals the confirmation of PPY-deposition (Fig. 4a). Fig. 3b and 4b show the C1s spectrum of blank and PPY-coated membrane. The deconvoluted C1s spectra of blank fabric show five peaks located at 285, 284.3, 288.7, 287.1, and 292.1 eV are attributed to C-O, C–C, C = O, C-CFx, and CF_2_ bonds, whereas, C-OH (285.1 eV), C = O (288.9 eV), C–C (284.4 eV), and C = N (286.4 eV) bonds appeared in PPY-coated fabric. Both O1s spectra of blank fabric (Fig. 3c) and PPY-coated fabric (Fig. 4d) show that the O element consists of C-OH (533.2 and 533.6 eV) and C-O-C (532.2 eV) bonds. Fig. 3d and 4e show flourine spectra composed of CF (689.5 eV and 684.2 eV) bond in blank fabric and PPY-coated fabric. The obvious N1s spectra have appeared only in PPY-coated fabric with N-H (401.1 eV) and C-N-C (399.8 eV) bonds. N1s spectra of PPY-coated confirm the well deposited PPY to the fabric surface [39], [40], [36], [41].

### 3.3 Contact angle measurement

**Fig. 5:**
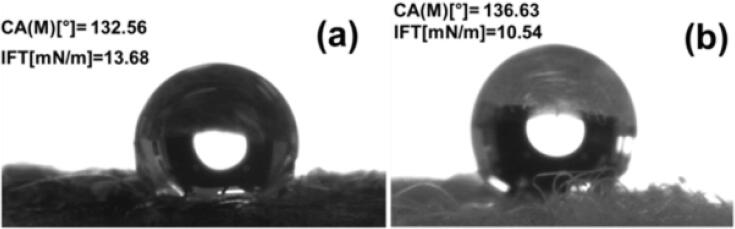
Water contact angle measurements of (a) PPY-fabric and (b) pristine fabric.

The surface wettability of the blank fabric and the PPY-coated fabric was assessed by water contact angle measurement. A drop of water on the pristine fabric was extremely spherical with a water contact angle (WCA) of 127°±7°, corroborating its hydrophobicity (Fig. 5a). When a water drop was placed on the PPY-coated fabric, the water was absorbed immediately, resulting in excellent hydrophilicity. Pristine fabric impeded the passage of both water and oil, whereas the PPY-coated fabric only allowed the passage of water. Hence, the PPY-coated fabric could be useful for oil/water separation and oil recovery.

### 3.4 Wettability

**Fig. 6:**
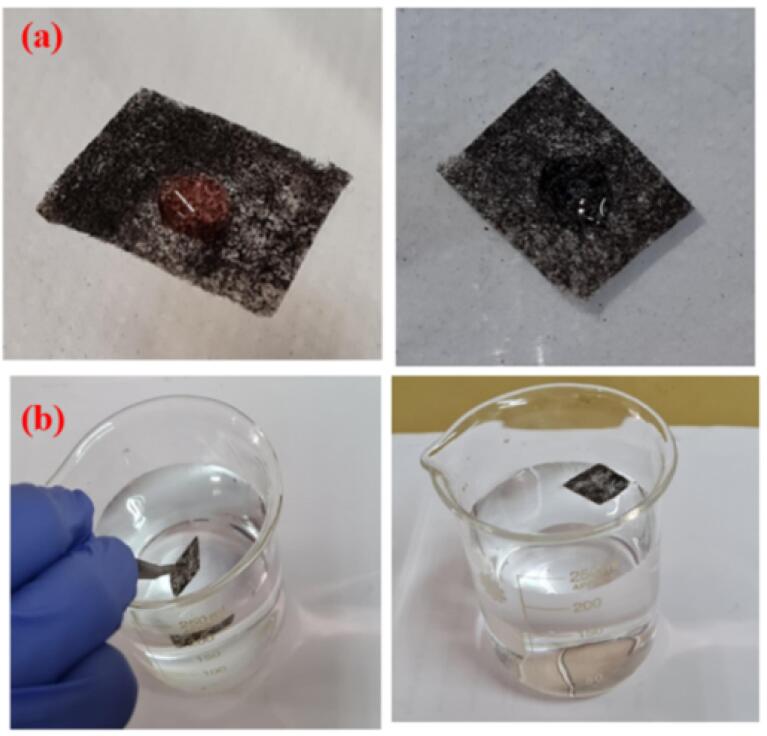
(a) Droplet-resistant test and (b) Wettability of the PPY-coated membrane.

The PPY coated fabric was immersed in water to visually examine its surface-wetting capability. After being dipped and soaking numerous times in water, the PPY coated material floating on the surface (Fig. 6b). Furthermore, water and oil droplets were deposited on the surface of the coated material. The oil droplet (red) remained on the surface, while water rapidly diffused into the fabric (Fig. 6a), illustrating that the coated surface can easily separate the oil from water contamination.

### 3.5 Oil absorption

**Fig. 7:**
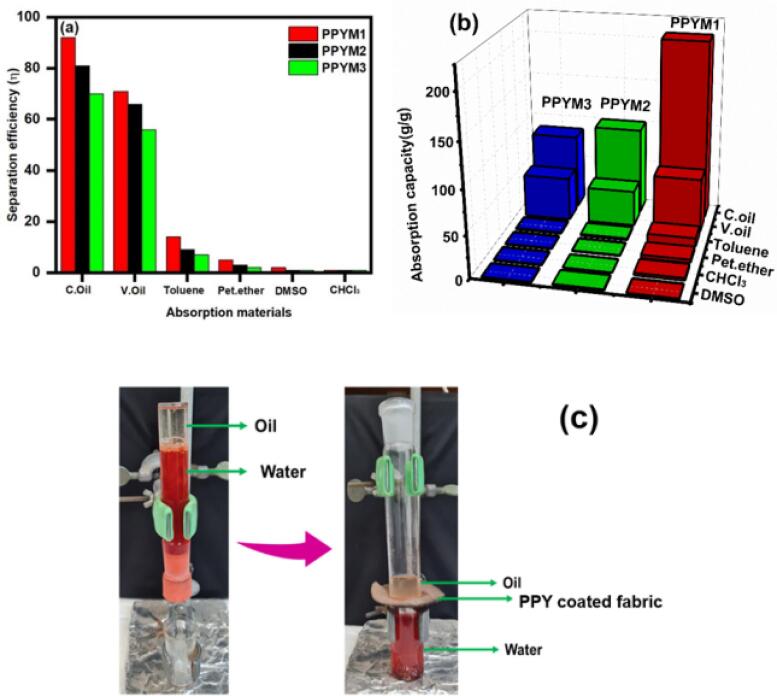
(a,b) The separation efficiency and absorption capacity of the PPY coated membrane and (c) the separation experimental setup for oil–water separation.‘-’: Not mentioned.

For water–oil separation and absorption, the PPY coated membrane exhibits good absorption capabilities. Other organic solvents and water mixtures were chosen as light and heavy oil absorption models, respectively, to examine the selective absorption ability of the PPY coated membrane toward oil and water. Various oils and organic chemicals were used in the absorption studies, including cooking oil, vacuum oil, toluene, petroleum ether, chloroform, and DMSO. The oil layer was absorbed by the PPY-coated membrane within 30 s of being soaked in the cooking oil/water mixture. Furthermore, significant oil absorption on the PPY coated membrane was due to the capillary force, which broke the trapped air layer between the material and water. The results show that the PPY membrane has high absorption of cooking oil and vacuum oil. The substance did not absorb all the other solvents entirely. In a comparable experiment without PPY, there was no difference in the absorption of oil by the uncoated material. The separation efficiency and absorption capacity for oil and other organic substances are shown in Fig. 7a, b, and Table 2. In brief, PPYM1 exhibited the strongest ability to absorb oil over all organic compounds. The enhanced absorption capacity could be attributed to the strong ultrasonic coating of PPY particles, which were well occupied on the membrane surface. Such results unraveled that only a small amount of ultrasonic energy was sufficient to anchor PPY particles on the membrane. Prolonged sonication might damage the membrane and/or delaminated PPY particles from the membrane. PPY particles reduce the surface energy and enhance the surface area for oil absorption as shown in Table 3.

**Table 2:** The absorption capacity of PPY

Absorption capacity (g)						
Materials	C. oil	V. Oil	Toluene	Pet. ether	CHCl_3_	DMSO
PPYM1	207	62	10	4	3	1
PPYM2	100	41	2	1	0.5	2
PPYM3	86	50	2	1	1.2	1.4

After oil separation, the spent PPY membrane was immersed in n-hexane to remove the absorbed oil to investigate the reuse of PPY materials. During the second and third cycles of the test, the PPY-coated fabric exhibited similar absorption. A lab-made setup consisting of an oil–water mixture tube (acted as a filter tube) with a dropper was designed to observe the high separation capacity of the PPY material (Fig. 7c). In this setup, the PPY coated fabric was placed between the beaker and the filter tube, containing a mixture of water and oil. When the filter tube was filled with an oil–water mixture, the PPY membrane only retained the oil but let the water flow through. The oil absorption efficiency of the PPY coated membrane was compared with the literature information (Table 3).

**Table 3:** The comparison table of oil separation efficiency with existing literature.

Adsorbent material	Fabrication method	Emulsion type	The amount of oil absorbed (%)	Ref.
PPy/Ag fabricPPy/Ag/OTS fabric	Gentle magnetic stirring	Water in chloroform emulsion	96.84	[36]
		Water in n-hexane emulsion	95.68	
		Methylbenzene in water emulsion	99.85	
PAN/PANI nanofiber	Electrospinning-Polymerization	Surfactant free-toluene in oil emulsion	99.8	[42]
1sBAT/PAN	Blending-Electrospinning	Surfactant free-hexane in oil emulsion	99.8	[43]
Au@ZIF-8@PAN-TD	Blending-Electrospinning-Surface modification	Surfactant stabilized oil-in-water emulsion	97.8	[44]
PAN/PS	Double layer electrospinning	Surfactant stabilized hexane-in-water emulsion	–	[45]
Laponite/CGN/h-PAN	Layer by layer modification	Surfactant stabilized n-hexadecane-in-water emulsion	> 99	[46]
PVA-SiO2/PDA/PEI	–	Surfactant stabilized oil-in-water microemulsion	99.5	[47]
PVP-TiO2 NPs	–	Gasoline-in-water emulsion	>99	[48]
PPY membrane	Ultra-sonication method	Cooking oil	93	This work
		Vacuum oil	81	
		Toluene	70	

### 3.6 Antibacterial activity

For the antibacterial activity, both bacterial strains (*E. coli* and *S. aureus*) were incubated overnight under aerobic conditions at 37 °C in Luria-Bertani (LB) broth. The bacterial concentration was measured using OD_595_ and a final concentration of 10^5^ bacteria was attained. For the antibacterial tests, 500 µL of the sample was added to the 500 µL of the bacterial suspension. After the mixture was incubated at 37 °C with shaking at 200 rpm, 100 mL aliquots were removed after 0, 12, and 24 h, diluted 10-fold in 20% LB medium and plated on nutrient agar plates. The materials were cut into small pieces (4*4 cm) and folded to prepare a multilayer for a precise antibacterial test. The multilayer material was then dipped into the bacterial solution for 24 h. The bacterial solution was serially diluted in a 96-well plate and plated on several agar plates.

**Fig. 8:**
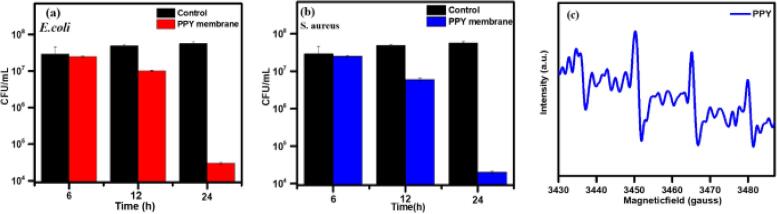
(a,b) Antibacterial activity of *E. coli* and *S. aureus* on PPYM and **(c)** EPR spectrum for the PPY.

The antibacterial activity was studied for the PPY membrane using the colony-forming per unit (CFU) method. The PPY membrane was treated against *E. coli* and *S. aureus.* Bacterial growth was checked for different incubation times to assess the killing ability and long-term activity of the PPY membrane. The bacterial test was conducted by dipping a multifold PPY membrane into the bacterial solution, followed by incubation at 6, 12, or 24 h. The PPY membrane was prepared to form a multilayer fold to absorb the bacterial solution completely and avoid the surface absorption of the bacterial solution. *E*. *coli* and *S*. *aureus* were subject to broad eradication after 24 h of exposure to the PPY membrane (Fig. 8a, b). The rationale behind the cell damage is the formation of free radicals by PPY, which was confirmed by electron paramagnetic resonance (EPR). For EPR analysis 5, 5-dimethyl-1-pyrroline-N-oxide (DMPO) was used as a spin trap to measure ROS generation. The spin trap material captures the OH radicals and superoxide anions to form DMPO-OH as a final product with high-intensity signals (Fig. 8c). The free radicals can interact with the cell membrane and control the cell growth activity on the surface. Oxidative DNA damage is well recognized as the primary cause of cell death. Moreover, the synergetic effect of the PPY was due to the free electrons in the polymer chain, which can be combined with oxygen in the solution to augment ROS production. Cell lysis could also be due to electrostatic and ionic interactions of the cell with abundant functional groups of the PPY ring. As the amine (NH) group of PPY with a positive charge readily interacts with negatively charged bacteria, leading to cell death. Detailed information on cell lysis can be found elsewhere [32], [33], [34], [35]. Biofouling is a serious problem for the use of membranes to process contaminated water as microorganisms adhere and grow on the membrane. They often produce extracellular polymers to impair the properties and efficiency of membranes. In this context, the PPY membrane with anti-biofouling is anticipated to process contaminated water, which is exposed to a broad range of microbes, including aerobic, anoxic, and anaerobic species. Fluorinated nonwoven materials exhibit some antimicrobial activities [49], however, this effect was not observed from the pristine membrane for both *E. coli* and *S. aureus* (control experiment).

## 4 Conclusion

In brief, the PPY material is successfully coated on the membrane using a simple one-step ultrasonication process. For the proper coating of PPY on the membrane, several ultrasonication times were used. An insignificant amount of the PPY is required for membrane coating and oil absorption. The ultrasonic approach can generate an excellent coating on the fabric or membrane. The developed PPYM material proved to be an effective adsorbent with selective absorption capability. The PPYM material could absorb various oils with an absorption capacity of 207 and 62 g/g, respectively, for the C and V oils. Furthermore, the antibacterial activity of the PPYM was investigated, with *E. coli* and *S. aureus* serving as two common model pathogens for the antibacterial test. The incorporation of PPY in fluorinated nonwoven fabric imparts antimicrobial activities against both Gram-negative and Gram-positive common pathogens.

## CRediT authorship contribution statement

**Moorthy Maruthapandi:** Conceptualization, Data curation, Methodology, Writing – original draft. **Arumugam Saravanan:** Data curation, Formal analysis, Methodology. **Shanmugasundaram Manoj:** Data curation, Software. **John H.T. Luong:** Writing – review & editing. **Aharon Gedanken:** Supervision.

## Declaration of Competing Interest

The authors declare that they have no known competing financial interests or personal relationships that could have appeared to influence the work reported in this paper.
